# Honeycomb Artifact Removal Using Convolutional Neural Network for Fiber Bundle Imaging

**DOI:** 10.3390/s23010333

**Published:** 2022-12-28

**Authors:** Eunchan Kim, Seonghoon Kim, Myunghwan Choi, Taewon Seo, Sungwook Yang

**Affiliations:** 1 Center for Intelligent and Interactive Robotics, Korea Institute of Science and Technology, Seoul 02792, Republic of Korea; 2 Department of Mechanical Convergence Engineering, Hanyang University, Seoul 04763, Republic of Korea; 3 Department of Biological Sciences, Seoul National University, Seoul 03080, Republic of Korea

**Keywords:** fiber bundle imaging, honeycomb artifact, pattern synthesis, convolution neural network (CNN)

## Abstract

We present a new deep learning framework for removing honeycomb artifacts yielded by optical path blocking of cladding layers in fiber bundle imaging. The proposed framework, HAR-CNN, provides an end-to-end mapping from a raw fiber bundle image to an artifact-free image via a convolution neural network (CNN). The synthesis of honeycomb patterns on ordinary images allows conveniently learning and validating the network without the enormous ground truth collection by extra hardware setups. As a result, HAR-CNN shows significant performance improvement in honeycomb pattern removal and also detailed preservation for the 1961 USAF chart sample, compared with other conventional methods. Finally, HAR-CNN is GPU-accelerated for real-time processing and enhanced image mosaicking performance.

## 1. Introduction

Fiber bundle endomicroscopy has great potential in in vivo, in situ diagnosis and interventional procedures in a wide range of clinical applications because of its flexibility and compactness [1]. For example, fiber bundle endomicroscopy has been deployed for optical biopsies predominantly in the gastrointestinal, urological and the respiratory tracts such as for cancer diagnosis [2,3,4]. However, images acquired through a fiber bundle suffer from artifacts such as honeycomb patterns, which are induced by void imaging space in the fiber bundle. Since the fiber bundle is composed of thousands of single optical fibers, the cladding layers of adjacent optical fibers inevitably block the light path. As a result, such void pixels in fiber bundle imaging degrade the quality of images and thus hinder accurate diagnosis.

A common remedy for the honeycomb artifact is to apply spatial or spectral filters onto acquired images because it is simple and fast enough for real-time processing. For the spatial filters, a Gaussian filter is commonly adopted to eliminate the artifact since it can smooth the edges of the single fibers in an efficient manner. For example, the Gaussian filter approach was introduced with histogram equalization for honeycomb pattern removal [5]. In addition to the Gaussian filtering, Winter et al. proposed an automatically generated adaptive mask in the spectral domain for filtering [6]. The use of convolution with a specifically designed kernel was also introduced for real-time processing in embedded systems [7]. Furthermore, Regeling et al. proposed spectral filtering for honeycomb pattern removal with minimal loss of information for cancer detection [8]. However, these approaches degrade the intensities of core pixels while smoothing the edges of honeycomb patterns. Accordingly, the resulting images become blurred.

Interpolation-based approaches can mitigate such a blurring effect as they preserve the intensities of the core pixels. They thus need to identify the locations of the core pixels prior to the interpolation, in which local maxima or circular Hough transforms are commonly used [9]. Natural neighbor interpolation can then be conducted with the Delaunay triangulation [10]. Wang et al. removed honeycomb patterns and mosaicked images using barycentric interpolation [11]. These approaches can be further improved. For example, Zheng et al. showed enhanced fiber bundle images by applying local binary patterns and nonlocal means subsequent to interpolation [12]. Although the interpolation-based methods can maintain the intensities of the core pixels, they involve preprocessing to identify core locations and interpolating gridded data, which are computationally expensive. Furthermore, these methods are only valid with the accurate detection of the core locations. Otherwise, resulting images are prone to distortion.

The superimposition of motion-induced images has been introduced for eliminating honeycomb artifacts and also enhancing limited resolution in fiber bundle imaging [13,14,15,16]. It recovers images by overlapping shifted images from successive frames, even without interpolation. Nevertheless, it is still challenging to apply these methods to real-time applications since multiple frames should be accumulated in acquisition and processing for image restoration.

Recently, deep learning-based approaches have also been proposed [17,18]. Shao et al. adopted a generative adversarial network (GAN) for image restoration [13]. To learn a direct mapping from fiber bundle to ground truth images, a dual-sensor fiber bundle imaging system was also developed, which offers ground truth data aligned with fiber bundle images. Later, a cascaded networks scheme improved restoration accuracy by utilization of multiple fiber bundle images [18]. A motion estimation network estimates unknown motions represented by homographies among successive fiber bundle images and a 3D convolution neural network then provide a mapping from aligned fiber bundle image sequences to their ground truth images. Since these supervised learning approaches demand ground truth data for training, the development of extra hardware setups is involved to obtain artifact-free images.

**Figure 1:**
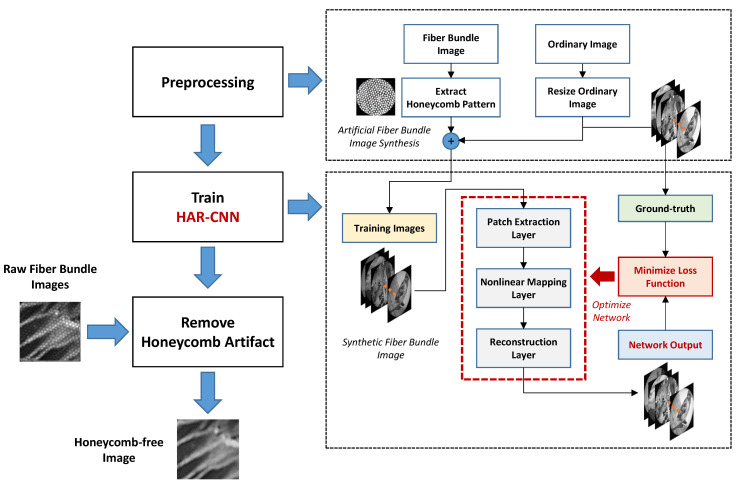
Workflow of HAR-CNN for honeycomb artifact removal in fiber bundle imaging.

In this paper, we propose a new deep learning framework for honeycomb artifact removal in fiber bundle imaging. The proposed framework offers an end-to-end mapping from given raw images to artifact-free images via a convolutional neural network regarded as a composition of nonlinear filters, as shown in Figure 1. Moreover, our reference-free approach introduces synthetic honeycomb patterns on ordinary images from an image database for training the network without an extra optical hardware setup for the ground truth collection. We validate the restoration performance of honeycomb-patterned images in terms of peak signal-to-noise ratio (PSNR) and structural similarity index measure (SSIM). In addition, the quality of honeycomb removal is evaluated on real fiber bundle images collected from a 1951 USAF resolution test chart. Finally, the proposed framework is GPU-accelerated for real-time processing and its honeycomb removal and mosaicking performances are also evaluated in imaging a lens tissue sample.

The main contribution of this paper is to provide a deep-learning based honeycomb artifact removal method that can be readily used for fiber bundle imaging. The honeycomb pattern synthesis eases the training procedure of the convolutional neural network with tremendous artificial data sets. In addition, the end-to-end mapping allows immediately restoring honeycomb-free images without any preprocessing upon their own honeycomb patterns, e.g., the number and location of fiber cores. In terms of performance, it shows significant performance improvement in honeycomb pattern removal and also detail preservation in real time, compared to the conventional methods.

Consequently, our approach tackles challenges in the conventional methods of honeycomb artifact removal. First, the deep-learning based method that can selectively removes honeycomb artifact overcomes the common limitation of spatial and spectral filtering, such as blurring both artifact and sample patterns. Second, the proposed method is robust to variation on the core locations of fiber bundles yielded occasionally by the reassembly or new uses of fiber bundles, which is prone to distortion in honeycomb removal with interpolation-based methods. Furthermore, the honeycomb-artifact synthesis scheme can lessen enormous burden raised by building extra hardware setups for the ground truth collection in other learning-based methods.

## 2. Materials and Methods

### 2.1. Deep Learning-Based Honeycomb Pattern Removal

#### 2.1.1. Training Dataset Generation via Honeycomb Pattern Synthesis

To learn the end-to-end mapping from raw images to artifact-free images, we utilize ordinary images for the ground truth from the image database (ImageNet Large Scale Visual Recognition Challenge 2013, ILSVRC 2013) [19] instead of using an extra hardware setup to simultaneously obtain artifact-free images. A honeycomb-like pattern is then synthesized on the ordinary images for training, where the pattern is taken from the white reference image of an actual fiber bundle. The white reference image thus indicates the core locations of the fiber bundle and void imaging area (honeycomb patterns) shown as bright and dark, respectively. Sequences for generating a training dataset are as follows. First, the ordinary images are converted to grayscale if they are colored images. The images are then resized corresponding to the pixel size of the imaging setup used; herein, we resize the images to 1024 × 1024 pixels. Next, we create a circular binary mask for a region of interest, where the outer region of the fiber bundle is forced to zero. Finally, pixel-wise multiplication is performed on the ordinary images with the white reference image. It is noted that the white reference image should be normalized between 0 to 1 to maintain the intensities of core pixels. These preprocessing steps are also illustrated in Figure 1.

#### 2.1.2. Deep Neural Network Architecture for Honeycomb Pattern Removal

The proposed deep learning framework adopts a convolutional neural network for honeycomb artifact removal. The proposed HAR-CNN (honeycomb-artifact-removal-convolution neural network) algorithm includes three convolution layers for image restoration: patch extraction, nonlinear mapping, and reconstruction. Accordingly, its architecture is similar to that of super-resolution CNN (SRCNN) [20]. The patch extraction layer creates a set of image patches encompassing the region of a single honeycomb. The nonlinear mapping layer is designed to deal with the non-linear features of honeycomb artifacts by transform high-dimensional vectors into low-dimensional vectors. Finally, the reconstruction layer outputs honeycomb-free images with high resolution by the convolution of neighboring pixels.

**Figure 2:**
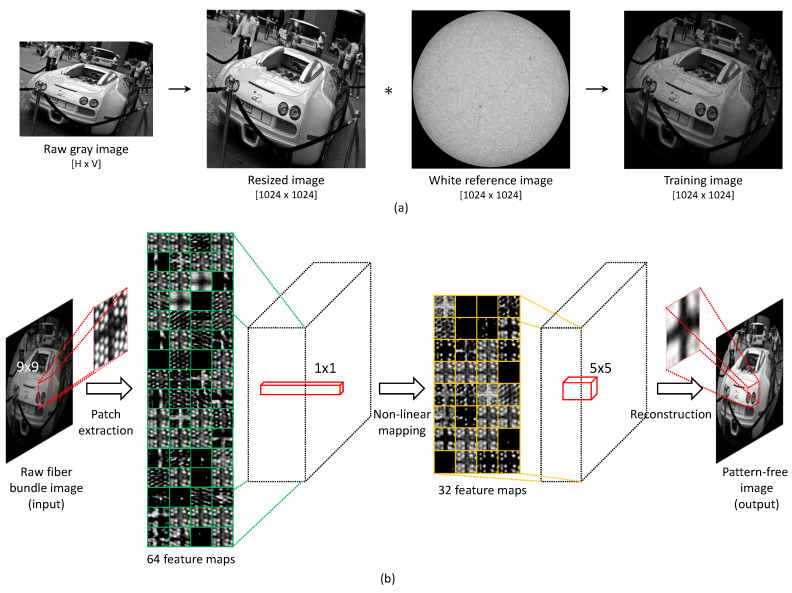
(**a**) Sequence of the training dataset generation via honeycomb pattern synthesis on ordinary images. (**b**) Network architecture of HAR-CNN with three convolution layers for patch extraction, nonlinear mapping, and reconstruction layers.

Since there is a trade-off between speed and performance according to the hyper-parameters of each layer, the appropriate spatial size of a kernel and the number of feature maps need to be set. For example, determining the filter size of the first layer is important to remove the honeycomb artifact. Through the investigation of the hyper-parameters, the first filter size is set to 9 to sufficiently enclose the honeycomb patterns because the distance between two adjacent cores is about 5–6 pixels with our imaging setup. The filter size of the nonlinear mapping layer was set to 1 with 64 feature maps, which provides enough nonlinear filtering and is also computationally efficient. Last, we adopt the filter size of the reconstruction layer as 5 with 32 feature maps. The entire network architecture is represented in Figure 2b.

The computational complexity of HAR-CNN is given by $O(\left(f_{1}^{2}n_{1}+n_{1}f_{2}^{2}n_{2}+n_{2}f_{3}^{2}\right)S_{HR})$, where $f_{i}$ and $n_{i}$ are the filter size and the number of filters at the *i*th layer, respectively, and $S_{HR}$ is the image size; $f_{1}=9$, $n_{1}=64$, $f_{2}=1$, $n_{2}=32$, and $f_{3}=5$.

To train HAR-CNN, a total of 130 images were used: 100 for training and 30 for validation. A single image is then decomposed into sub-images with a size of 33 × 33 and with a stride of 14, which creates 5184 sub-images in total. Thus, the total 518,400 sub-images were used for training. The batch size was set to be 128 and mean squared error (MSE) was used as a loss function. The activation function in the first and the second layers was adopted as rectified linear units (ReLU). The network model was trained with the stochastic gradient descent optimizer with a learning rate of 1e-4 for 10,000 epochs using TensorFlow. Image restoration performance for the number of training epoch will be further discussed in the Result section. Finally, the proposed algorithm was implemented on a C/C++ platform with an OpenCV library and accelerated by the use of a graphical processing unit (GPU, Geforce RTX 3090, NVIDIA) for real-time processing. As a result, the honeycomb removal is accomplished within 10 ms for a raw fiber bundle image with a 1024 × 1024-pixel size.

### 2.2. Experimental Setup

**Figure 3:**
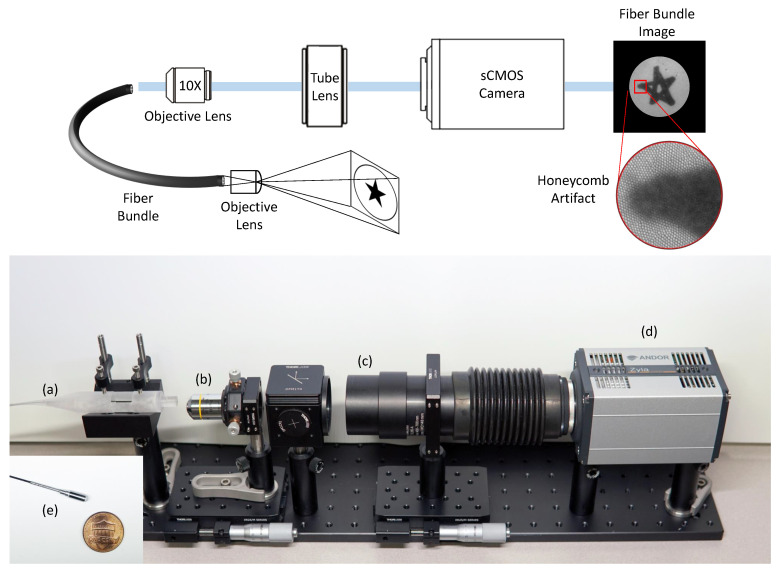
Hardware setup for the fiber bundle imaging system. (**a**) the proximal end of the fiber bundle, (**b**) objective lens (10×), (**c**) tube lens, (**d**) sCMOS camera, and (**e**) the distal end of the fiber bundle.

Our fiber bundle imaging setup consists primarily of a fiber bundle (FIGH-30-650S, Fujikura Ltd., Tokyo, Japan), an 10X objective lens (RMS10X, 0.25NA, 10.6 mm WD, Olympus Corp., Tokyo, Japan), a tube lens (TTL200-A, Thorlabs Inc., Newton, NJ, USA), and an sCMOS camera (Zyla 4.2 PLUS, Oxford Instruments, Abingdon, UK) as shown in Figure 3. The fiber bundle includes 30,000 fiber cores with melted silica claddings in a 650-μm diameter. The distal end of the fiber bundle is coupled with an aspheric lens with a 3-mm diameter (#15-271, Edmund Optics Inc., Barrington, NJ, USA) for enlarging field-of-view, which provides a field of view of 2.2 mm at 20-mm apart from a target. The incident light propagates through the fiber bundle and is imaged at the sCMOS camera with a pixel resolution of 1024 × 1024 at 100 Hz for high-speed imaging.

## 3. Results

### 3.1. Validation of HAR-CNN on Synthetic Images

We validated the restoration performance of HAR-CNN on synthetic images by superimposing artificial honeycomb patterns on original images, which are taken as the ground truth for quantitative evaluation. The quantitative evaluation was performed with two metrics–peak signal-to-noise ratio (PSNR) and structural similarity index measure (SSIM) widely used for quality evaluation in image restoration or enhancement. PSNR is to quantify restoration quality for images while comparing pixel-wise intensity difference between two images as in Equation (1).

(1) $$PSNR\left(dB\right)=20\log\left(\frac{255}{MSE}\right),$$

where MSE is the mean squared error between the ground truth and estimated image. SSIM is adopted as a perceptual metric that quantifies image quality degradation compared to the ground truth, as a combination of three independent components, luminance, contrast, and structure [2].

(2) $$SSIM=\frac{\left(2\mu_{x}\mu_{y}+c_{1}\right)\left(2\sigma_{xy}+c_{2}\right)}{\left(\mu_{x}^{2}+\mu_{y}^{2}+c_{1}\right)\left(\sigma_{x}^{2}+\sigma_{y}^{2}+c_{2}\right)}$$

(3) $$c_{1}={\left(0.01L\right)}^{2}\;\mathrm{and}\;c_{2}={\left(0.03L\right)}^{2},$$

where $\mu_{x}$, $\mu_{y}$, $\sigma_{x}$, $\sigma_{y}$ and $\sigma_{xy}$ are the local means, standard deviations, and cross-covariance for images *x*, *y*. *L* is a dynamic range of pixel values.

**Figure 4:**
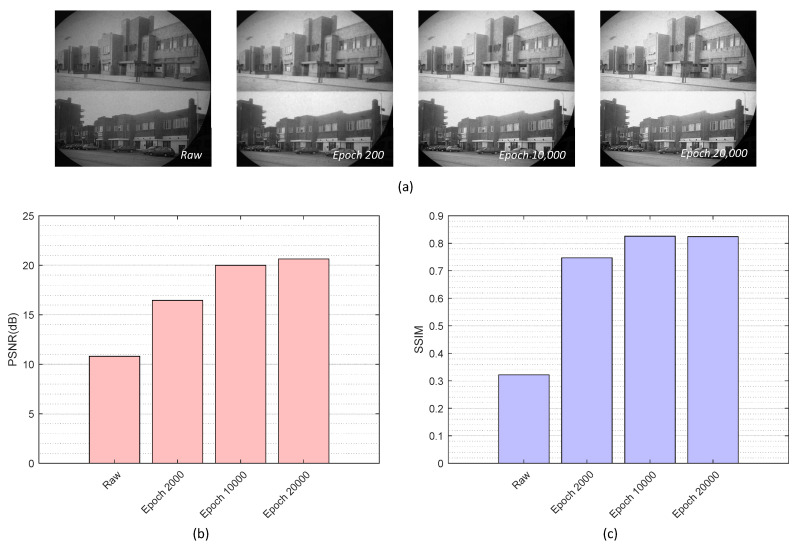
(**a**) Restored images for various training epochs, (**b**) PSNR according to the increment of training epoch, (**c**) SSIM according to the increment of training epoch.

**Figure 5:**
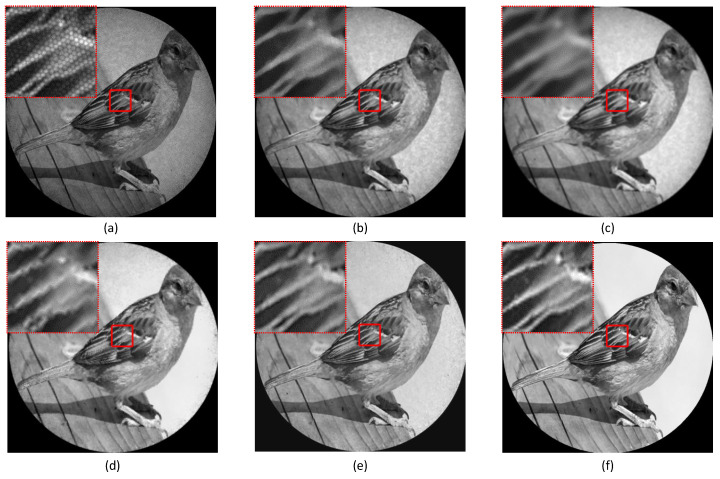
Honeycomb artifact removal with various methods. (**a**) Synthetic fiber bundle image (raw), (**b**) median filter, (**c**) Gaussian filter, (**d**) interpolation, (**e**) HAR-CNN, and (**f**) ground truth image.

First, we examined image restoration performance of HAR-CNN along with the increment of training epochs—2000, 10,000, 20,000 using PSNR and SSIM. As shown in Figure 4, there was no significant improvement between 10,000 and 20,000 epochs in image restoration. The restoration performance of HAR-CNN was then compared with other conventional methods: median filter, Gaussian filter, and the interpolation method in [11]. The sizes of the applied filters were set to 7 × 7 and 3 × 3, for the median and Gaussian filters, respectively. Figure 5 shows qualitative results of honeycomb pattern removal on a synthetic fiber bundle image. It is found that HAR-CNN most preserves the detail of the image after restoration as shown in a magnified region of Figure 5e (see bird’s feather). In addition, we obtained the highest PSNR with HAR-CNN. It indicates that the proposed network was appropriately trained subject to minimizing MSE between output images and the corresponding ground truth. SSIM is also highest with HAR-CNN compared to the other methods. Interestingly, the median filter’s SSIM score is highest among the conventional methods while the interpolation method’s PSNR score is higher than that of the median filter. This is because the median filter could further preserve the sharpness of the edges compared to the other conventional methods. The quantitative results are summarized in Table 1.

### 3.2. Evaluation of Honeycomb Pattern Removal on 1951 USAF Target

**Figure 6:**
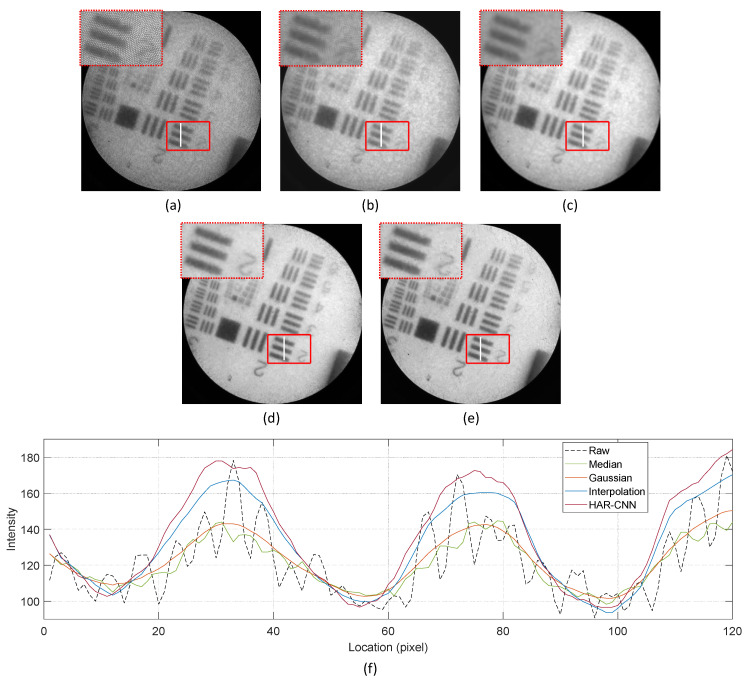
Resulting images for various honeycomb removal methods on the USAF target sample: (**a**) raw fiber bundle image, (**b**) median filter, (**c**) Gaussian filter, (**d**) interpolation, (**e**) HAR-CNN. (**f**) Pixel intensities along a line of interest shown as a white line within the line-patterned region, where the values are averaged across a column width of two.

We evaluated the proposed method on real fiber bundle images taken on a 1951 USAF target. Figure 6 shows the resulting images for various honeycomb pattern removal algorithms.

An inset figure at the top-left corner of each resulting image shows the magnified view of a cropped region with a red square in order to present restoration details. We also investigated the intensity values along a line of interest marked as a white line in the region. As a result, significant intensity modulations are observed in the raw image because of void imaging pixels as shown in Figure 6f. These wavy modulations become diminished as the filtering methods, interpolations, and the proposed HAR-CNN are applied. As shown in the magnified view, the edges of lines are clearly distinguishable with the interpolation method and HAR-CNN while those are still fuzzy with the median and Gaussian filtering because of a smoothing effect.

For further quantitative analysis, two quality measures, variance-based smoothness and Rayleigh-based line separation criteria are introduced. First, the variance-based smoothness describes how well the honeycomb effect is mitigated and smoothed. The smoothness, *s* is defined by the ratio between the variances of a raw fiber bundle image and a restored image at a flat region on the USAF target as in Equation (4).

(4) $$s=1-\frac{\sigma_{s}}{\sigma_{0}}=1-\sqrt{\frac{\sum_{i,j\in R_{s}}{\left(s_{s}^{image}\left(i,j\right)-\mu_{s}\right)}^{2}}{\sum_{i,j\in R_{s}}{\left(s_{0}^{image}\left(i,j\right)-\mu_{0}\right)}^{2}}},$$

where σ${}_{s}$ and μ${}_{s}$ are the standard deviation and mean value of pixel intensities $s_{s}^{image}\left(i,j\right)$ at the region of interest, $R_{s}$ in the restored image. $\sigma_{0}$ and $\mu_{0}$ are calculated from the raw fiber bundle image in the same manner. Accordingly, smaller variance at the restored image leads to higher smoothness. In contrast, the Rayleigh-based line separation criterion, *r* was adopted to evaluate how each algorithm preserves details after removing the honeycomb patterns, which is thus calculated in a line-spaced region, $R_{r}$. An averaged intensity value $S_{l}\left(k\right)$ along a specified line $R_{r}^{row}$ is obtained by Equation (5).

(5) $$S_{l}\left(k\right)=\frac{1}{M}\sum_{j\in R_{r}^{col}}s_{r}^{image}\left(k,j\right)\;\mathrm{for}\;k\in R_{r}^{row},$$

where $R_{r}^{row}$ and $R_{r}^{col}$ contain row and column pixel coordinates, respectively. $s_{r}^{image}\left(k,j\right)$ is a pixel value at the *k*th row and *j*th column and *M* is the number of columns in the region $R_{r}$. Finally, the line separation quality *r* is defined as in Equation (6).

(6) $$r=\frac{\max\left(S_{l}\left(k\right)\right)-\min\left(S_{l}\left(k\right)\right)}{\max(S_{l}\left(k\right))}$$

To take both honeycomb removal performance and detail preservation into account, we also investigated a weighted quality measure *q* by combining these two metrics, *s* and *r*, with a weighting factor γ.

(7) q=γ·s+(1−γ)·r

The weighting factor thus represents the importance of honeycomb pattern removal compared to detail preservation.

We compared the performance of the HAR-CNN with the conventional honeycomb pattern removal methods. The identical filter sizes adopted in synthetic images evaluation were also used for the median and Gaussian filtering methods. The interpolation method is also the same as that used for synthetic image restoration. According to the variance-based smoothness, HAR-CNN shows the best honeycomb removal performance and it is followed by the Gaussian filter. In addition, the image details are also most preserved with HAR-CNN as the line-separation quality measure is above 0.45. For the weighted quality measure *q*, the highest scores are also obtained with HAR-CNN for both the weighting factor, 0.5 and 0.8. The quality measures for each algorithm are summarized in Table 2.

### 3.3. Honeycomb Pattern Removal and Image Mosaicking on Lens Tissue Sample

**Figure 7:**
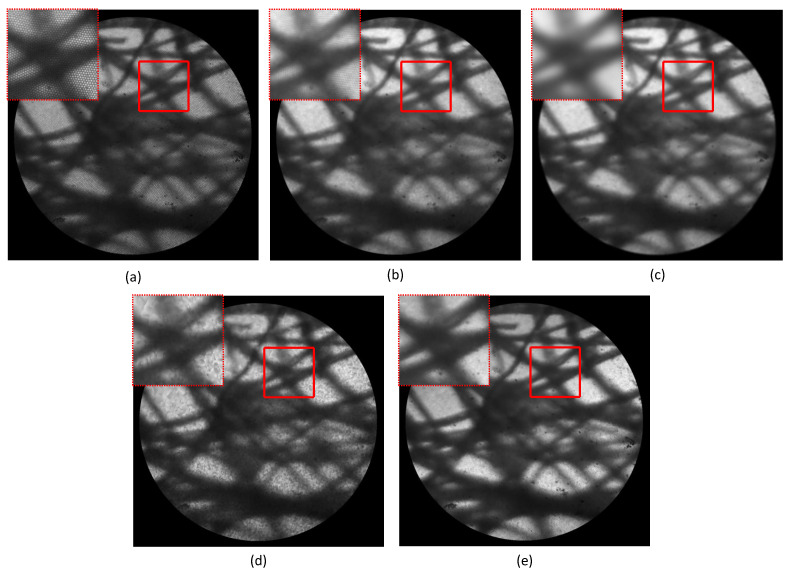
Resulting images for various honeycomb removal methods on the lens tissue sample: (**a**) raw fiber bundle image, (**b**) median filter, (**c**) Gaussian filter, (**d**) interpolation, and (**e**) HAR-CNN (see Video S1). The red-squared region is magnified at the top-left corner of each image to clearly show image restoration details.

We also evaluated the performance of HAR-CNN on a lens tissue sample. To visualize the fibrous structure of the lens tissue, the test was conducted without the objective lens located at the distal end of the fiber bundle, which yields a field-of-view (FOV) of 650 μm in diameter. Image mosaicking is then accompanied to obtain an enlarged image from such a small FOV. A common method for image mosaicking is to first extract feature points (e.g., distinct corner points) and then match their correspondences in a subsequent frame. Therefore, it is necessary to eliminate honeycomb patterns to successfully achieve image mosaicking in fiber bundle imaging. Otherwise, the stationary and distinct honeycomb patterns are recognized highly likely as image features instead of shifted sample images. Consequently, detection of such spurious feature points would hinder accurate image mosaicking. The resulting images for the various honeycomb removal methods on the lens tissue sample are shown in Figure 7. As found in the earlier section, HAR-CNN clearly restored the details of the fibrous structures while efficiently eliminating honeycomb patterns.

**Figure 8:**
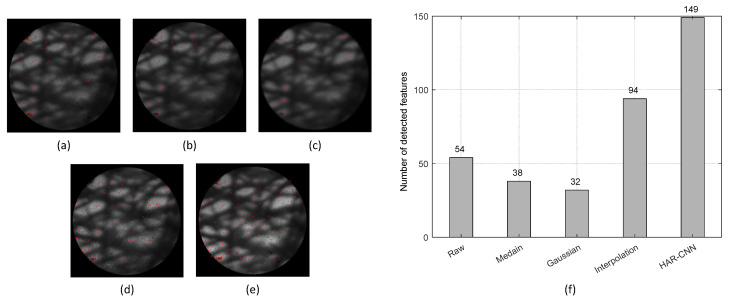
Detected image feature points for various honeycomb removal methods on the lens tissue sample: (**a**) raw fiber bundle image, (**b**) median filter, (**c**) Gaussian filter, (**d**) interpolation, and (**e**) HAR-CNN. The red cross indicates a detected image feature point. (**f**) The number of detected image features for the various honeycomb removal methods.

Prior to image mosaicking, we also investigated the number of image features found in a restored image by each algorithm. As shown in Figure 8, HAR-CNN provides more numbers of image features compared to the other methods. Interestingly, the raw image gives more image features compared to the spatial filtering methods. However, it should be noted that some of these detected image features could be rather spurious, which may lead to inaccurate image mosaicking as presented in Figure 8b.

**Figure 9:**
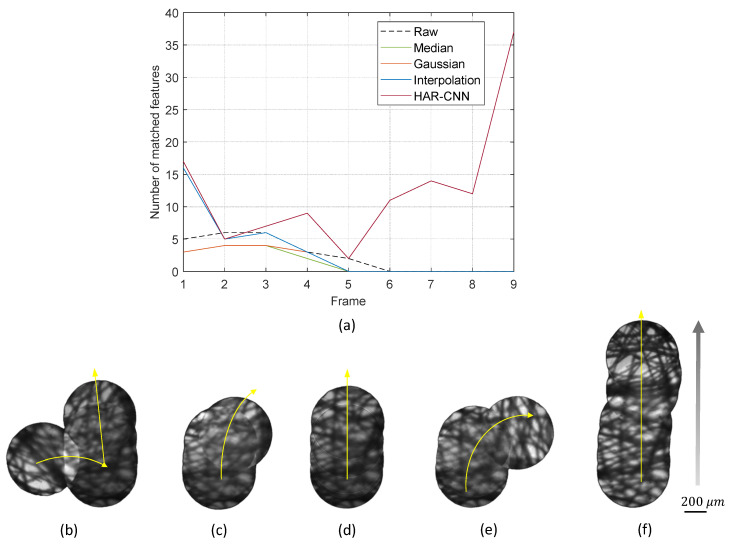
(**a**) The number of matched image features between successive frames for various honeycomb removal methods. (**b**–**f**) Mosaic images during a handheld scan of the fiber bundle probe on the lens tissue sample: (**b**) by use of raw images, (**c**) median filter, (**d**) Gaussian filter, (**e**) interpolation, and (**f**) HAR-CNN for honeycomb pattern removal.

Image mosaicking performance was evaluated during a handheld scan of the fiber bundle probe along an upward direction for a length of approximately 1.7 mm. As a result, the proposed HAR-CNN yielded a vertically well-aligned mosaic image, since it provided a sufficient number of image features for accurate correspondence matching, as shown in Figure 9f. On the other hand, mosaicking results of the other methods were either fairly limited or distorted. For example, mosaic results by the median filtering and interpolation were distorted as is the result of the raw fiber bundle images due to spurious feature points. In the Gaussian filtering, the lack of extracted features impeded further image mosaicking although a well-aligned mosaic image was obtained only at the beginning of the scan.

## 4. Discussion

We proposed a deep learning framework to eliminate honeycomb artifacts via a convolutional neural network with the artifact synthesis scheme on the ordinary image dataset. Hence, the proposed method utilizes a single white-reference image that contains honeycomb patterns for artifact synthesis. It provides the ground truth for training the network in an efficient manner because the ordinary images themselves can be taken as the ground truth without extra hardware setups to obtain enormous ground-truth images. As a result, our HAR-CNN offers an end-to-end mapping from a raw fiber bundle image to a honeycomb-free image.

Although the white-reference image taken from a single fiber bundle was used for the artifact synthesis, the trained network was able to be applied to other new fiber bundles regardless of their own honeycomb patterns. We also found that HAR-CNN was robust to the translational and/or rotational variations of honeycomb patterns, which could occasionally occur during the installation of the fiber bundle. For example, no loss in image restoration was observed while the restoration performance by the interpolation-based method was significantly degraded regarding the alignment variation by the reassembly of the fiber bundle. This is because the CNN algorithm can be treated as a set of nonlinear convolutional filters on a raw fiber bundle image. Accordingly, the proposed algorithm could seamlessly deal with the reassembly or replacement of fiber bundles without any modification of the trained network.

The experimental results show that HAR-CNN significantly improves the honeycomb removal performance compared to the conventional methods while preserving image details. In a perspective of the application of fiber bundle imaging, the proposed method is superior to the other conventional methods in image feature extraction and matching, which is important for post-processing of fiber bundle images such as for image mosaicking. Furthermore, the GPU acceleration of the proposed algorithm allows a real-time image restoration even for the large size of a fiber bundle image. The image restoration with HAR-CNN is completed within 10 ms for a raw fiber bundle image with a 1024 × 1024-pixel size (100 fps), while conventional filtering methods still take about 3–4 ms for the same image size. Compared to other similar networks, HAR-CNN showed reliable performance in image restoration and also time-efficient processing. For example, honeycomb removal adopting enhanced deep residual networks (EDSR) [21] with the same dataset is prone to saturation in relatively bright images and also takes 0.9–1.0 s per a frame, which is unsuitable for real-time application.

The comparison with the other deep learning-based methods [13,14] in terms of PSNR and SSIM is not available yet. Since our hardware setup is designed for generic fiber bundle imaging, the ground truth data for such evaluation is not offered. Regarding these measures, our results for the synthetic images are, however, comparable to those results by the other deep learning-based methods. Since HAR-CNN was trained with the single white reference image from a specific fiber bundle, its application to various fiber bundle models could potentially be limited. Accordingly, further improvement could also be made by adopting a multi-scale/level feature learning scheme in order to deal with a large variety of fiber bundles with different core distances through a unified network structure. In addition, the combination of other deep neural networks may further improve its restoration performance and enhance fundamentally limited resolution in fiber bundle imaging. Future plans include testing the proposed algorithm in imaging tissue samples in vivo.

## Figures and Tables

**Table 1:** Image restoration performance of various algorithms for honeycomb-pattern synthesized image.

Method	Raw	Median ^1^	Gaussian ^2^	Interpolation	HAR-CNN
PSNR (dB)	20.58	19.74	18.98	23.66	26.52
SSIM	0.7301	0.7978	0.6878	0.7719	0.9119

^1^ Size of median filter is 7 × 7 and ^2^ standard deviation of Gaussian filter is 3.

**Table 2:** Comparison of quality measures for various honeycomb removal methods.

Method	*s* ^1^	*r* ^2^	*q* ^3^ (γ=0.5)	q(γ=0.8)
Raw image	0	0.4654	0.2327	0.0931
Median	0.6610	0.2880	0.4745	0.5864
Gaussian	0.7725	0.2821	0.5273	0.6744
Interpolation	0.7509	0.4044	0.5886	0.6992
HAR-CNN	0.7758	0.4569	0.6164	0.7120

^1^ Variance-based smoothness, ^2^ Rayleigh-based line separation criteria, and ^3^ weighted quality measure with a weighting factor γ.

## Data Availability

Not applicable.

## Supplementary Materials

The following supporting information can be downloaded at: https://www.mdpi.com/article/10.3390/s23010333/s1, Video S1: Real-time demonstration of HAR-CNN on the lens tissue sample.
